# Automated Radiology Alert System for Pneumothorax Detection on Chest Radiographs Improves Efficiency and Diagnostic Performance

**DOI:** 10.3390/diagnostics11071182

**Published:** 2021-06-29

**Authors:** Cheng-Yi Kao, Chiao-Yun Lin, Cheng-Chen Chao, Han-Sheng Huang, Hsing-Yu Lee, Chia-Ming Chang, Kang Sung, Ting-Rong Chen, Po-Chang Chiang, Li-Ting Huang, Bow Wang, Yi-Sheng Liu, Jung-Hsien Chiang, Chien-Kuo Wang, Yi-Shan Tsai

**Affiliations:** 1 Department of Medical Imaging, National Cheng Kung University Hospital, College of Medicine, National Cheng Kung University, No. 1 University Road, Tainan 704, Taiwan; n102225@mail.hosp.ncku.edu.tw (C.-Y.K.); smygbrew@hotmail.com (C.-C.C.); n100435@mail.hosp.ncku.edu.tw (H.-S.H.); n100358@mail.hosp.ncku.edu.tw (H.-Y.L.); n103777@mail.hosp.ncku.edu.tw (C.-M.C.); n105705@mail.hosp.ncku.edu.tw (K.S.); n103068@mail.hosp.ncku.edu.tw (T.-R.C.); n104306@mail.hosp.ncku.edu.tw (P.-C.C.); leatinghuang@gmail.com (L.-T.H.); i54941386@gs.ncku.edu.tw (B.W.); taicheng100704@yahoo.com.tw (Y.-S.L.); n625396@mail.hosp.ncku.edu.tw (C.-K.W.); 2 Department of Medical Imaging, E-DA Hospital, I-Shou University, No. 1 Yida Road, Jiaosu Village, Yanchao District, Kaohsiung 824, Taiwan; ed112930@edah.org.tw; 3 Department of Computer Science and Information Engineering, College of Electrical Engineering and Computer Science, National Cheng Kung University, No. 1 University Road, Tainan 704, Taiwan; jchiang@mail.ncku.edu.tw

**Keywords:** pneumothorax, artificial intelligence, deep learning, Radiology Alert System

## Abstract

We aimed to set up an Automated Radiology Alert System (ARAS) for the detection of pneumothorax in chest radiographs by a deep learning model, and to compare its efficiency and diagnostic performance with the existing Manual Radiology Alert System (MRAS) at the tertiary medical center. This study retrospectively collected 1235 chest radiographs with pneumothorax labeling from 2013 to 2019, and 337 chest radiographs with negative findings in 2019 were separated into training and validation datasets for the deep learning model of ARAS. The efficiency before and after using the model was compared in terms of alert time and report time. During parallel running of the two systems from September to October 2020, chest radiographs prospectively acquired in the emergency department with age more than 6 years served as the testing dataset for comparison of diagnostic performance. The efficiency was improved after using the model, with mean alert time improving from 8.45 min to 0.69 min and the mean report time from 2.81 days to 1.59 days. The comparison of the diagnostic performance of both systems using 3739 chest radiographs acquired during parallel running showed that the ARAS was better than the MRAS as assessed in terms of sensitivity (recall), area under receiver operating characteristic curve, and F1 score (0.837 vs. 0.256, 0.914 vs. 0.628, and 0.754 vs. 0.407, respectively), but worse in terms of positive predictive value (PPV) (precision) (0.686 vs. 1.000). This study had successfully designed a deep learning model for pneumothorax detection on chest radiographs and set up an ARAS with improved efficiency and overall diagnostic performance.

## 1. Introduction

Chest radiography is the most common first-line imaging examination for the screening of multiple thoracic diseases, [1] including pneumothorax, a potentially life-threatening condition requiring clinical attention [2]. The mean sensitivity of detecting pneumothorax by upright chest radiography varies from 80–86% in different studies [3,4].

Recent advancements in machine learning have promising results in various fields of radiology. An accuracy of 86% for detecting pneumothorax has been reached in a study based on the “ChestX-ray8” database of frontal chest radiographs with disease labels [5]. In another study, a deep-learning algorithm for multiple thoracic diseases on chest radiographs derived from single-center data, the accuracy of detecting pneumothorax reached 95% [6].

In the current scenario of busy radiology practice, examinations containing critical findings are frequently hidden in the long worklist of reporting radiologists. An automated detection system based on machine learning may screen these examinations for critical findings, notify the referring primary care physicians, and flag these examinations to be read by radiologists as early as possible [7]. Current studies regarding the application of machine learning in radiology have focused on the accuracy of their machine learning algorithms, but no study has revealed the time an automated detection system based on machine learning could save in the real-world setting.

At our institution, Radiology Alert System for pneumothorax detection on chest radiographs had been online since 2015. The existing Radiology Alert System was operated by the radiologic technologists in charge and is termed as “Manual” Radiology Alert System (MRAS) in this article. In this study, an Automated Radiology Alert System (ARAS) of pneumothorax detection on chest radiographs was set up by a deep learning model, and then the efficiency and diagnostic performance of the MRAS and ARAS were compared at the tertiary medical center.

## 2. Materials and Methods

### 2.1. Patients and Image Acquisition

Institutional Review Board approval was obtained, and the requirement to obtain informed consent was waived. A retrospective search of the Picture Archiving and Communication System (PACS) and the MRAS for chest radiographs with pneumothorax alerts from 1 January 2015 to 31 December 2019, and another retrospective search of the PACS and the Radiology Information System (RIS) for chest radiographs with “pneumothorax” in the reports from 1 January 2013 to 31 December 2014 (before the MRAS went online) were performed and found 2090 chest radiographs with pneumothorax, among which 570 radiographs were excluded due to negative pneumothorax, age less than or equal to 6 years, poor positioning, poor image quality, metallic implants masking lung fields, and chest wall deformity.

The remaining radiographs were classified according to the extent of pneumothorax by 6 radiologic technologists and reviewed by a 4th year radiology resident. Pneumothorax classified as “small” demonstrated up to 1 cm separation between visceral and parietal pleura that was confined to one area of the lung (e.g., apex, lateral, medial, base). Pneumothorax classified as “moderate” revealed greater than 1 cm separation between visceral and parietal pleura and were confined to one region of the lung, or up to 2cm separation between visceral and parietal pleura and involved more than one area of the lung. Pneumothorax classified as “large” showed greater than 2 cm separation between the parietal and visceral pleura and involved more than one area of the lung. Among the 1520 chest radiographs, 285, 377, and 858 were classified as small, moderate, and large pneumothorax, respectively. Images classified as “small” pneumothorax were not used in the training [8]. Totally 1235 radiographs with moderate and large pneumothorax were subjected to polygonal segmentation of the area of pneumothorax by 6 radiologic technologists and reviewed by a 4th year radiologist resident using Labelme [9].

**Figure 1:**
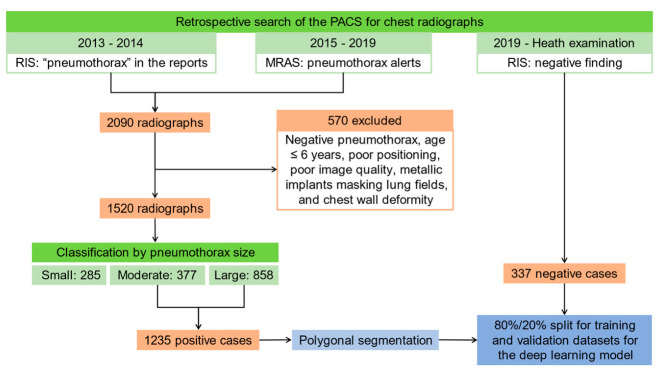
The flow chart of the data acquisition for the deep learning model for pneumothorax detection on chest radiographs. Abbreviations: PACS, Picture Archiving and Communication System; MRAS, Manual Radiology Alert System; RIS, Radiology Information System.

As for negative cases, another retrospective search of the PACS and RIS for chest radiographs for health examination with negative findings in 2019 was performed and 337 chest radiographs were identified. The positive cases with segmented areas of pneumothorax and negative cases were shuffled and separated into an 80%/20% split for training and validation datasets for the deep learning model. The flow chart of the data acquisition for the deep learning model for pneumothorax detection on chest radiographs is shown in Figure 1.

### 2.2. Deep Learning Model

**Figure 2:**
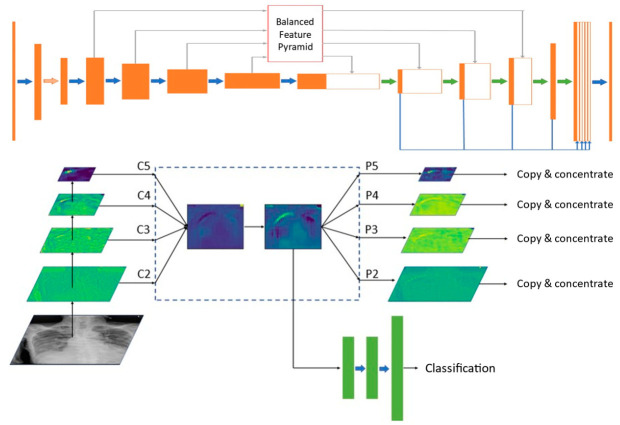
The overall pneumothorax detection network and the pipeline and heatmap visualization of the balanced feature pyramid module.

To achieve the goal of detecting pneumothorax, a well-designed model was proposed based on U-Net with ResNet34 as an encoder [10,11]. Utilizing multi-level features to generate discriminative pyramidal representations was crucial to detection performance. Our model utilized balanced feature pyramids in skip connections and integrated multi-level contextual features in the decoder [12]. The overall pneumothorax detection network and the pipeline and heatmap visualization of the balanced feature pyramid module are shown in Figure 2. The design of the deep learning model is described in Appendix A.

### 2.3. Manual and Automated Radiology Alert Systems

**Figure 3:**
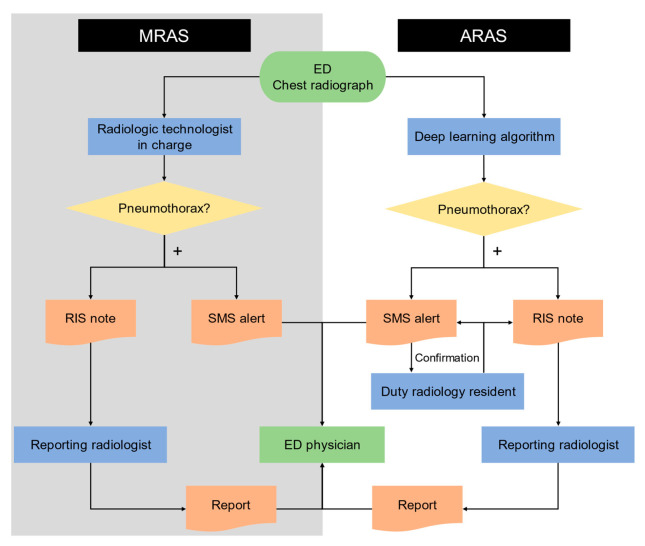
The flow charts of the manual radiology alert system and the automated radiology alert system. Abbreviations: MRAS, Manual Radiology Alert System; ARAS, Automated Radiology Alert System; SMS, Short Message System; RIS, Radiology Information System.

Both the MRAS and ARAS for pneumothorax were applied to the Emergency Department (ED). In the MRAS, the radiologic technologists in charge of taking the chest radiographs would alert the ED physicians via Short Message System (SMS) and then leave notes in the RIS notifying reporting radiologist if pneumothorax were newly detected. Newly detected pneumothorax was defined as those without prior radiographs showing pneumothorax and without pigtail or chest tube drainage. In the ARAS, the deep learning model described previously would alert the ED physicians via SMS and leave notes in the RIS notifying reporting radiologist if pneumothorax were detected. The SMS alerts were also sent to duty radiology residents for confirmation. The flow charts of both systems are shown in Figure 3.

### 2.4. Efficiency of Deep Learning Model

From 21 July 2015 to 13 May 2019, the alerts sent from the MRAS for pneumothorax on chest radiographs acquired in the ED were retrospectively collected. After the ARAS went online, from 1 September 2020 to 31 October 2020, all chest radiographs acquired in the ED of our institution were subjected to the ARAS for pneumothorax, and the alerts sent from the ARAS in this period were collected. In both systems, the image upload time was assigned as the starting point, and the alert time and the report time were recorded. The mean alert time and mean report time of both alert systems were compared.

### 2.5. Diagnostic Performance during Parallel Running of the Two Systems

From 1 September 2020 to 31 October 2020, a parallel running strategy of both MRAS and ARAS for pneumothorax was implemented at our institution. Both systems operated simultaneously and independently. The radiologic technologist in the MRAS did not know the detection result of the ARAS, and the deep learning model in the ARAS had no additional input other than the chest radiograph itself. Chest radiographs acquired in the ED with an age of more than 6 years during this period were prospectively used as the testing dataset for both systems. These radiographs were reviewed by a 4th year radiology resident and a radiologist with more than 10 years of working experience and classified as negative, small, moderate, and large pneumothorax. Since the MRAS was activated only if pneumothorax were newly detected while all pneumothorax detections activated the ARAS, the two systems were compared on an equal basis. An alert sent from the MRAS on one of the serial radiographs with pneumothorax would be interpreted as positive detections for all these radiographs. Then the confusion matrices of both MRAS and ARAS were obtained. The false-positive cases of the ARAS were also reviewed, and the incorrectly predicted areas of the deep learning model and possible undesirable conditions were recorded.

### 2.6. Statistical Analysis

Analyses were conducted using Microsoft Excel 2016 MSO (Microsoft, version 16.0.13426.20308) and MedCalc (MedCalc Software, version 19.5.6). The alert time and the report time in the MRAS and ARAS were compared using an unpaired *t*-test. As for diagnostic performance, the sensitivity (recall), specificity, positive predictive value (PPV) (precision), negative predictive value (NPV), accuracy, area under receiver operating characteristic (ROC) curve (AUC), and F1 score along with their 95% confidence intervals were calculated for comparison.

## 3. Results

### 3.1. Deep Learning Model

**Figure 4:**
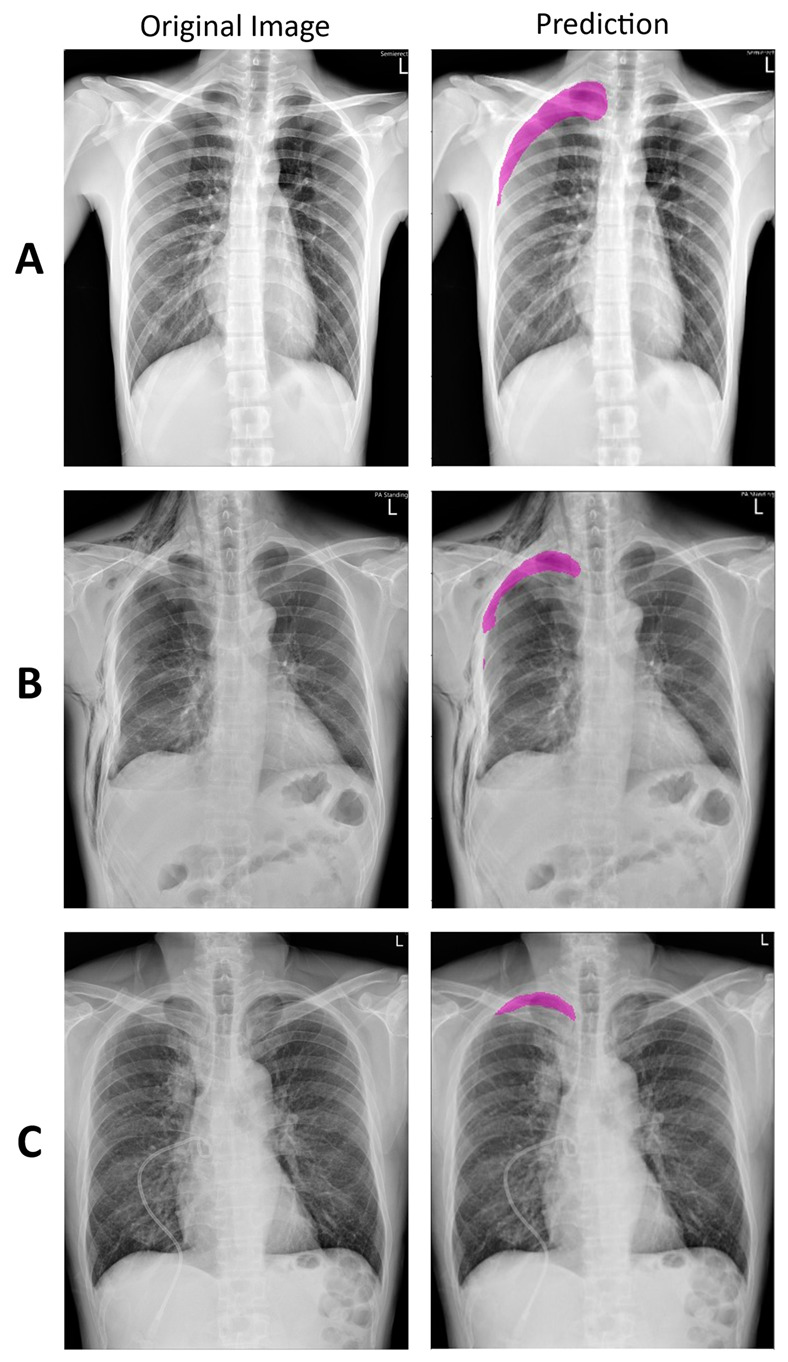
Examples of true-positive detections of large (**A**), moderate (**B**), and small (**C**) pneumothorax. The predicted areas are shown in purple.

In this study, 1235 positive cases with segmented areas of pneumothorax and 337 negative cases were shuffled and separated for training and validation datasets for the deep learning model. The training datasets contained 979 positive cases and 278 negative cases, and the validation dataset contained 256 positive cases and 59 negative cases. A deep learning model based on U-Net with balanced feature pyramid modules for pneumothorax detection was built using the training and validation datasets stated above. Then, the model was used to set up the new ARAS. Examples of true-positive detections of pneumothorax are shown in Figure 4.

### 3.2. Efficiency of Deep Learning Model

From 21 July 2015 to 13 May 2019, 217 pneumothorax alerts were sent from the MRAS for chest radiographs acquired in the ED. The mean alert time and the mean report time of the MRAS were 8.45 min (range, 0.02–160.35 min) and 2.81 days (range, 0.01–10.33 days), respectively. From 1 September 2020 to 31 October 2020, 105 pneumothorax alerts were sent from the ARAS for chest radiographs acquired in the ED. The mean alert time and the mean report time of the ARAS were 0.69 min (range, 0.22–2.20 min) and 1.59 days (range, 0.01–6.81 days), respectively. Both the mean alert time (*p*-value < 0.0001) and the mean report time (*p*-value < 0.0001) were significantly shorter in the ARAS than in the MRAS.

### 3.3. Diagnostic Performance during Parallel Running of the Two Systems

During parallel running of both the MRAS and the ARAS for pneumothorax from 1 September 2020 to 31 October 2020, 3739 chest radiographs were acquired in the ED with an age greater than 6 years. After retrospective review by radiologists, 86 chest radiographs were found to have pneumothorax, among which 13, 20, and 53 were classified as small, moderate, and large pneumothorax, respectively. During this period, nine alerts for a new detection, interpreted as 22 detections after review, were sent from the MRAS, and 105 alerts were sent from the ARAS. The confusion matrices of the MRAS and ARAS are shown in Table 1. The sensitivity (recall), specificity, PPV (precision), NPV, accuracy, and AUC, along with their 95% confidence intervals, of the MRAS and ARAS are shown in Table 2. The sensitivity (recall) was significantly lower in the MRAS than in the ARAS (0.256 vs. 0.837). The sensitivity (recall) for small, moderate, and large pneumothorax in the ARAS were 0.615 (8/13), 0.700 (14/20), and 0.943 (50/53), respectively. A smaller extent of the pneumothorax was associated with lower sensitivity (recall) in the ARAS. The PPV (precision) was significantly higher in the MRAS than in the ARAS (1.000 vs. 0.686). The AUC was significantly lower in the MRAS than in the ARAS (0.628 vs. 0.914), with *p*-value < 0.0001. The F1 score was lower in the MRAS than in the ARAS (0.407 vs. 0.754). Both MRAS and ARAS demonstrated high specificity, NPV, and accuracy (greater than 0.98), with slightly higher specificity and slightly lower NPV in the MRAS than in the ARAS with statistical significance (1.000 vs. 0.991 and 0.983 vs. 0.996, respectively) and the similar accuracy in both systems without significant difference (0.983 vs. 0.987). In summary, the diagnostic performance of both systems during parallel running showed that the ARAS was better than the MRAS in terms of sensitivity (recall), AUC, and F1 score, but worse in terms of PPV (precision).

**Figure 5:**
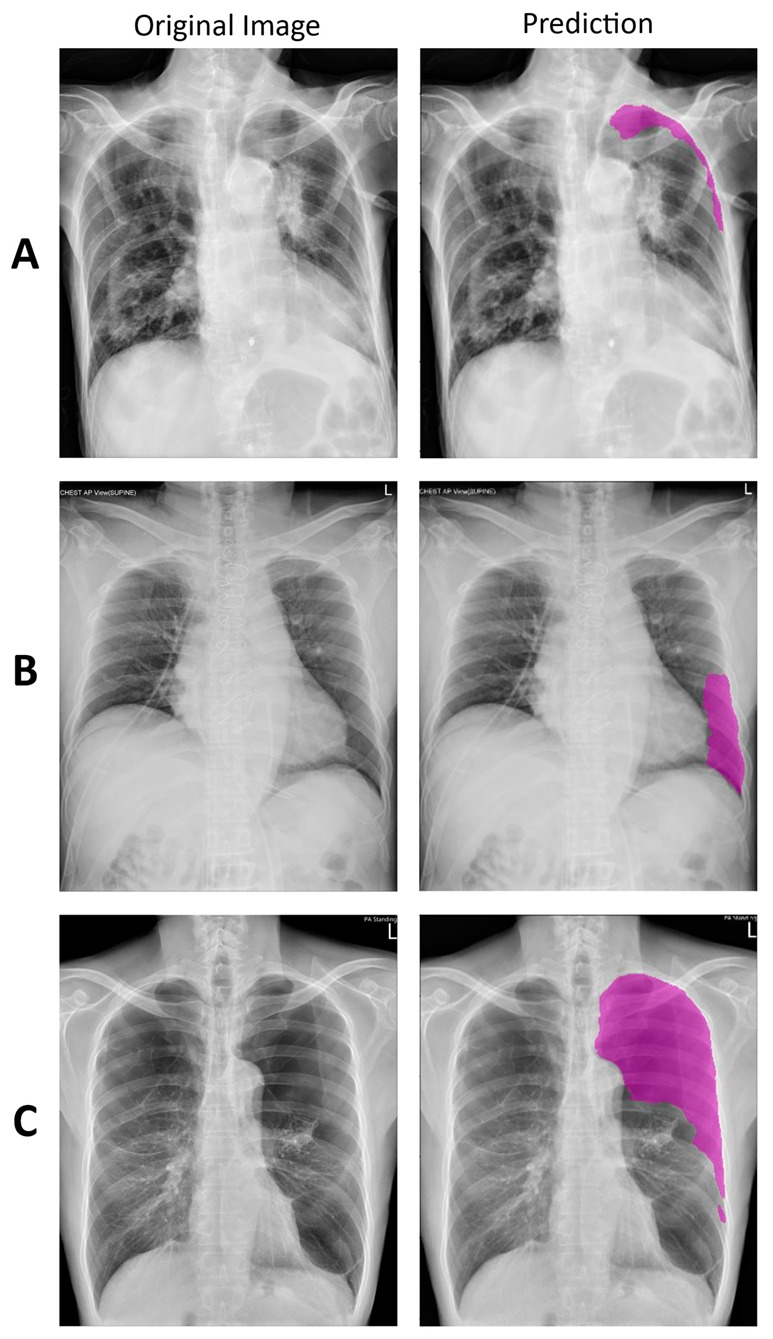
Examples of false-positive detections of pneumothorax with predicted area along ribs (**A**), heart border (**B**), and bullae (**C**). The predicted areas are shown in purple.

A review of the 33 false-positive cases with the predicted areas of the deep learning model was performed. The incorrectly predicted area included 23 at the lung apex along the ribs, aortic arch, or thickened pleura; 3 at the lung base along the heart border or ribs; 3 with bullae; 2 along the skin fold; 1 detecting gastric gas; and 1 along the chest tube. The possible undesirable conditions included eight with foreign bodies (metallic implants, chest tubes, or central venous catheters), one with poor positioning, two with poor exposure settings, and one with severe lung fibrosis were identified. Examples of false-positive detections of pneumothorax are shown in Figure 5.

## 4. Discussion

In this study, the efficiency of detecting pneumothorax was improved after using the deep learning model. The mean alert time was significantly shorter in the ARAS than in the MRAS. In the MRAS, the radiologic technologists in charge could see the chest radiographs firsthand even before the images were uploaded. However, the high workload distracted radiologists with little time spent viewing the radiograph, delaying both pneumothorax detections and the subsequent alert activation. In the ARAS, the deep learning model could “view” the radiographs almost immediately after image upload and send alerts soon after pneumothorax detections without interference by the working environment. The ARAS with a mean alert time of 0.69 min (or 41.4 s) with a maximum of 2.20 min (or 132.0 s) provided warnings in a constantly efficient manner.

In both MRAS and ARAS, the reporting radiologists received the notes in the RIS about pneumothorax detections and then completed the reports with high priority. However, the mean report time was significantly shorter in the ARAS than in the MRAS in this study, which might result from the following two factors. Firstly, shorter mean alert time in the ARAS than in the MRAS implied leaving notes in the RIS earlier for the reporting radiologists. Secondly, the reporting radiologist received not only RIS notes from the deep learning model, but also confirmation messages sent from duty radiology residents in the ARAS. This reconfirmation process might have given the reporting radiologists stronger suggestions to complete the reports earlier.

During the parallel running of both systems, the sensitivity (recall) was significantly lower in the MRAS than in the ARAS (0.256 vs. 0.837). High workload causing distraction in the MRAS might have resulted in low sensitivity (recall), while the ARAS was not interfered by the working environment. Both MRAS and ARAS demonstrated specificity, NPV, and accuracy greater than 0.98, with minor or no difference statistically. As for the overall performance, the ARAS outperformed the MRAS in terms of AUC and F1 score. The performance of the deep learning model implemented in the ARAS, with the sensitivity (recall) of 0.837, the specificity of 0.991, the accuracy of 0.987, and AUC of 0.914, was good and comparable to previous studies [6,8,13,14,15,16].

During parallel running of both systems, the PPV (precision) was significantly higher in the MRAS than in the ARAS (1.000 vs. 0.686). A review of the false-positive cases showed that 78.8% (26/33) of the cases had incorrectly detected normal anatomical border (ribs, aortic arch, thickened apical pleura, or heart border), while other “stripes” (skin folds or chest tube), and lucency (bullae or gastric gas) were also misleading. The performance of the model might be even worse with foreign bodies, poor positioning, or poor exposure settings. A high false-positive rate of the ARAS posed a major problem of clinical use. Frequent false alarms would have caused the “crying wolf” phenomenon, and the ED physicians would be less willing to pay attention to the alerts. A confirmation process by the duty radiology resident was added to the system to compensate for this issue and would remain necessary until further improvement of the deep learning model.

This study aimed to design an alert system for potential emergencies, especially with large pneumothorax as indication for chest tube drainage, while small pneumothorax is often treated conservatively. In this study, only moderate to large pneumothorax was subjected to training, while small pneumothorax was excluded. The same criteria were used in a previous study [8]. In the testing dataset, some cases of small pneumothorax were still detected by the ARAS, with a smaller extent of the pneumothorax associated with lower sensitivity (recall). The sensitivity (recall) of the ARAS for moderate and large pneumothorax reached 0.877 (64/73).

This study excluded patients with an age of less than 6 years. This consideration was mainly due to the epidemiology of pneumothorax. The earliest peak in the age distribution of spontaneous pneumothorax is 15 to 20 years [17,18,19]. Spontaneous pneumothorax is extremely rare in preschoolers. On the other hand, preschool patients are often not cooperative with instructions to position and hold breath, leading to the poor image quality of the radiographs and subsequent misinterpretation of the deep learning model. Excluding patients with age less than 6 years allowed the ARAS to focus on the population at risk.

Several issues resulted in the limitations of this study. First of all, the ground truths of radiographs in both training and validation datasets were based on the interpretation of radiologists. Misclassification was still possible even after reviewing process. Secondly, the datasets for training, validation, and testing were relatively small compared to other studies [6,8,15,16,20]. Expansion of the datasets from our institution or using public datasets should be considered. Thirdly, the deep learning model tended to be interfered with by foreign bodies, poor positioning, and poor exposure settings, which were not uncommon in daily practice. Training of the model for chest radiographs with these undesirable conditions might solve this problem. Finally, the ARAS still needed a confirmation process due to the high false-positive rate. Further improvement of the deep learning model was crucial to make this system fully “automated”.

## 5. Conclusions

This study has successfully designed a deep learning model for the detection of pneumothorax on chest radiographs and set up an ARAS. The efficiency of detecting pneumothorax was improved after using the deep learning model. During the parallel running of both systems, the diagnostic performance of the ARAS was better than that of the MRAS in terms of sensitivity (recall), AUC, and F1 score, but worse in terms of PPV (precision).

## Figures and Tables

**Table 1:** Confusion matrices of the Manual Radiology Alert System and Automated Radiology Alert System for pneumothorax.

			MRAS				ARAS					
			Positive		Negative		Positive		Negative		Total	
Ground Truth	Positive	Small	22	2	64	11	72	8	14	5	86	13
		Moderate		3		17		14		6		20
		Large		17		36		50		3		53
	Negative		0		3653		33		3620		3653	
	Total		22		3717		105		3634		3739	

Note: MRAS = Manual Radiology Alert System, ARAS = Automated Radiology Alert System.

**Table 2:** Diagnostic performance of Manual Radiology Alert System and Automated Radiology Alert System for pneumothorax.

	MRAS	ARAS
Sensitivity (Recall)	0.256 (0.168–0.361)	0.837 (0.742–0.908)
Specificity	1.000 (0.999–1.000)	0.991 (0.987–0.994)
PPV (Precision)	1.000 (1.000–1.000)	0.686 (0.605–0.756)
NPV	0.983 (0.981–0.985)	0.996 (0.994–0.998)
Accuracy	0.983 (0.978–0.987)	0.987 (0.983–0.991)
AUC	0.628 (0.612–0.643)	0.914 (0.905–0.923)
F1 score	0.407 (0.391–0.423)	0.754 (0.740–0.768)

Note: Data are reported as value with 95% confidence interval in parentheses. MRAS = Manual Radiology Alert System, ARAS = Automated Radiology Alert System, PPV = positive predictive value, NPV = negative predictive value, AUC = area under receiver operating characteristic curve.

## Data Availability

The data presented in this study are available on request from the corresponding author.

## Appendix A

The U-Net architecture has been widely used in medical imaging tasks as it shows promising results [10]. U-Net uses the concepts of skip connections and joints convolutional layers with pooling layers and up-sampling layers to create contractive and expansive paths. Skip connections are implemented to leverage intermediate feature maps and merge contractive and expansive features. Different from U-Net, which integrates multi-level features using the lateral connection, we strengthen the multi-level features using the same deeply integrated balanced semantic features. Each resolution in the pyramid obtains equal information from others, thus balancing the information flow and leading the features more discriminative. Specifically, we first resized the multi-level feature (C2, C3, C4, C5) to an intermediate size. Then the balanced semantic features are obtained by averaging as follows:

(A1) $$C=\frac{1}{L}\overset{l_{max}}{\underset{i=l_{min}}{\sum }}\hat{C}i$$

where *L* denotes the number of multi-level features, $l_{min}$ and $l_{max}$ denote the indices of involved lowest and highest levels, and ${\hat{C}}_{i}$ denotes respective rescaled features. The obtained features are then rescaled using the reverse procedure to strengthen the original features.

We further implemented the attention module in each stage of encoder, Convolutional Block Attention Module (CBAM), to improve the representation of interests [21]. The CBAM module is a lightweight and general module, it can be plugged into any CNN architecture with negligible overheads. The CBAM module applies two attention modules consecutively. The first attention module is applied channel-wise, in that we want to select the features that are independent of spatial ones. The second attention module is applied along the spatial dimensions to select the features which are more relevant to each other and independent of the channels. Both modules generate attention maps, respectively, then the attention maps are multiplied to the input feature map for adaptive feature refinement.
